# Asymptomatic Right Ventricular Hypoplasia in Twin Siblings: A Normal Variant or Cause of Early Mortality?

**DOI:** 10.1155/2019/6871340

**Published:** 2019-01-21

**Authors:** Amna Qasim, Soham Dasgupta, Ashraf M. Aly

**Affiliations:** ^1^Dept. of Pediatrics, University of Texas Medical Branch, Galveston, TX, USA; ^2^Dept. of Pediatrics, Emory University School of Medicine, Atlanta, GA, USA; ^3^Division of Pediatric Cardiology, University of Texas Medical Branch, Galveston, TX, USA

## Abstract

Right ventricular (RV) hypoplasia may develop secondary to pulmonary or tricuspid valve atresia. These patients are usually symptomatic early in life and need prompt intervention. Isolated RV hypoplasia is a rare congenital heart disease. We report a case of 23-year-old twins who have been monitored for the last 14 years for isolated right ventricular hypoplasia. ECHO and MRI studies showed a small, heavily trabeculated, nonapex-forming RV and mild tricuspid valve insufficiency. The girl has a patent foramen ovale (PFO). Otherwise, the cardiac anatomy and function was normal. They have both been completely asymptomatic from the cardiac standpoint. The family history is remarkable for death of father at the age of 30 years with autopsy suggestive of a hypoplastic RV. The paternal uncle also died at the age of 46 years, and his son has an unidentified congenital heart disease. The family history appears to suggest an autosomal dominant pattern of inheritance with variable expressivity. However, the chromosome microarray analysis of the twins did not identify any variations of clinical significance.

## 1. Introduction

Isolated right ventricular hypoplasia (IRVH) is a rare congenital heart disease that is different from the severe right ventricular hypoplasia that is commonly described with tricuspid or pulmonary valvular defects [1–8]. There are a few case reports of IRVH associated with a small atrial septal defect/patent foramen ovale (ASD/PFO) found in patients of different ages who presented with cyanosis and/or signs of congestive heart failure [3]. The familial nature of this defect has also been seen in the reported cases [9, 10]. However, IRVH occurring in completely asymptomatic siblings has not been described before. We describe a set of twins with IRVH who are currently completely asymptomatic from the cardiovascular standpoint.

## 2. Case Report

Nine-year-old twin siblings (a boy and a girl) were initially referred to our clinic by their new primary care pediatrician who was concerned because the twins' father died from an unknown heart condition and possibly had a small right ventricle seen on autopsy (documentation not available). Their paternal uncle also died at the age of 46 years from a cardiac cause (details unknown), and his son has an unidentified congenital heart disease.

**Figure 1:**
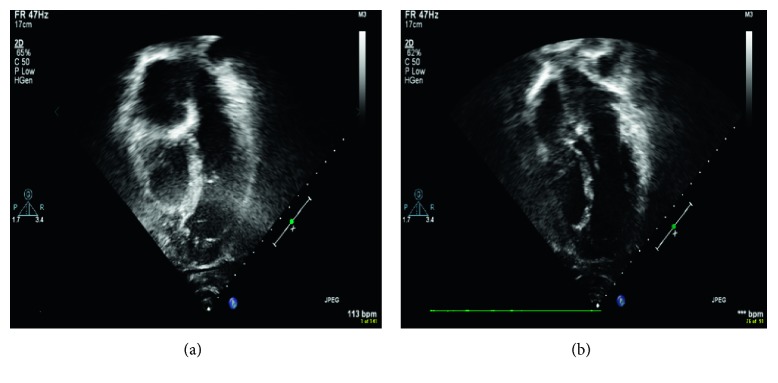
Four-chamber echo showing RV hypoplasia in twin boy (a) and girl (b).

At initial presentation, the twins were both asymptomatic from a cardiac standpoint. Cardiac exam revealed no murmurs or gallops, and the EKG was normal. Echocardiogram showed mild to moderate hypoplasia of the right ventricle with normal wall thickness which was not apex forming. The distance from the tricuspid valve annulus to the lowermost portion of the right ventricle was 4.6 cm and 4.5 cm in the boy and girl, respectively, while the distance from the mitral valve annulus to the true apex was 8.4 cm and 7.7 cm, respectively (Figure 1). The left ventricle was relatively dilated and had normal function. The girl had a patent foramen ovale (PFO). There were no other cardiac anomalies found. The *z*-scores of tricuspid valve annulus were +0.77 and +0.04 for the boy and girl, respectively, and the *z*-scores of the pulmonary valve annulus were −1.49 and −1.44, respectively.

Since their initial visit, we continued to follow them closely every year. The girl had occasional episodes of dizziness and irregular heart beats; a Holter monitor showed a few premature ventricular complexes (PVCs) that disappeared during an exercise stress test. The boy had a normal EKG and stress test.

**Figure 2:**
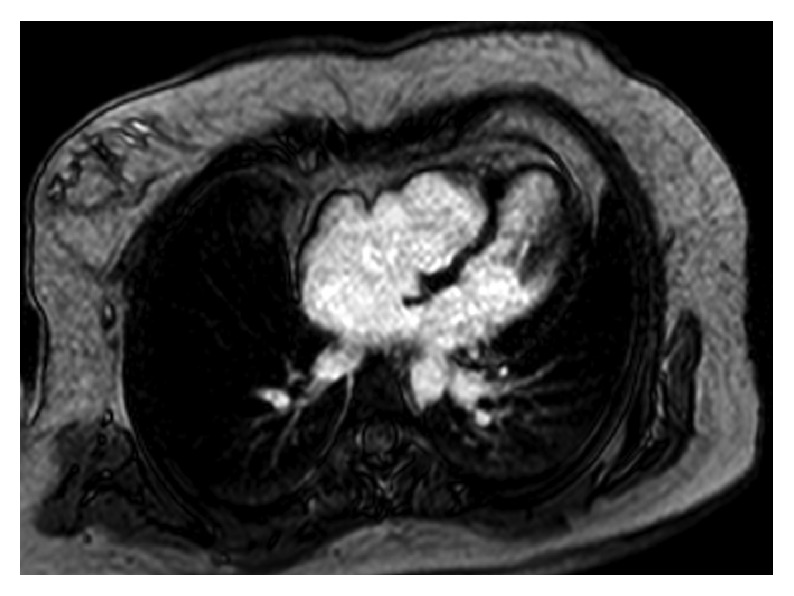
Nonapex-forming RV seen on MRI in twin boy.

The siblings are now 23 years old and have been doing well from a cardiovascular standpoint. Due to the concerns of early death of the father, a cardiac MRI was performed which revealed a nonapex-forming right ventricle with a small size of trabecular portion (Figure 2). The effective right ventricular volume was normal. Indexed right ventricular end-diastolic volume was 76 cc/m^2^, and RV ejection fraction was 51%. No MRI evidence of arrhythmogenic right ventricular dysplasia, right ventricular outflow tract aneurysm, or myocardial scarring was noted. MRI on the girl revealed similar findings.

Chromosome microarray analysis of the twins did not identify any copy number variations that are of clinical significance based on the current reporting criteria.

## 3. Discussion

The RV is considered hypoplastic if the distance from the tricuspid valve annulus to the RV apex is less than half the distance from the mitral valve annulus to the LV apex [11]. Severe forms of hypoplasia present in infancy and are usually associated with obstructive lesions of the tricuspid or pulmonary valves that lead to a reduction in blood flow to the RV. RV hypoplasia may also develop secondary to Ebstein's anomaly of tricuspid valve, absence of the right coronary artery, anomalies of systemic venous return, Bernheim's syndrome (displacement of interventricular septum), or congenital absence of the right ventricular myocardium leading to a thin parchment-like ventricular wall (Uhl's anomaly) [3]. RV hypoplasia in adults is often secondary to disruption in the blood supply to the right ventricular myocardium due to a coronary artery anomaly [12]. IRVH is described in the absence of these conditions when the right ventricular cavity size is reduced and the ventricular wall thickness is normal. Most cases in literature of IRVH are associated with an ASD or a PFO.

The zinc finger transcription factors GATA4 and GATA6 have been suggested to play critical roles in embryonic myocardial development. Mice embryos that are heterozygous for GATA4 and GATA6 alleles were found to have a complex spectrum of cardiovascular defects including thin-walled myocardium, ventricular septal defects, and abnormal smooth muscle defects [13]. Transgenetic studies have shown that the heart tube is initially composed of the prospective left ventricular myocardium, and the right ventricular myocardial cells are added on anteriorly from the anterior heart field [14]. Mutations in transcription factors or improper signaling of migratory cells may play a role in the development of IRVH.

In a case series that summarized fifteen cases of IRVH, all the cases were associated with either an ASD/PFO and all presented with cyanosis. Most of these cases were found to have right atrial enlargement and a right-to-left shunt through the interatrial defect. Seven out of the fifteen cases were reported to be familial [3]. Chessa et al. reported IRVH in a 1-day-old male and his 34-year-old father with no significant finding on genetic testing [9].

We describe a rare case of IRVH in a set of asymptomatic twins. Even though the right ventricle is hypoplastic, it is able to maintain sufficient pulmonary blood flow based on echocardiographic and MRI studies. MRI imaging of the right ventricle integrates volumetric and functional analysis in a comprehensive approach, including assessment of cardiac morphology, myocardial tissue characteristics, and flow patterns [15]. Despite the small size of the RV in the cardiac MRI in our patients, it was still able to generate adequate ejection fraction, which may explain the lack of symptoms. Surgical repair of IRVH was reported in a case series of 4 patients and involved closure of ASD with a right atrium (RA) to pulmonary artery (PA) conduit. The postoperative course was complicated by arrhythmias and/or sinus node dysfunction in all the patients, and the authors concluded that the RA to PA connection or Fontan's repair should be restricted for patients with severe hypoplasia [16].

Even though the family history appears to suggest an autosomal dominant pattern of inheritance with variable expressivity, chromosomal microarray did not identify any variants of clinical significance. It is possible that the genes playing a role in the pathogenesis of this condition have not yet been identified.

We assume that IRVH in the absence of other cardiac anomalies is likely to be a normal variant in heart development and does not need any medical/surgical intervention in the absence of symptoms. To our knowledge, no other cases of completely asymptomatic familial IRVH have been reported.

## 4. Conclusion

IRVH in asymptomatic patients may be a normal variation in cardiac development. It is prudent to follow these patients clinically along with serial echocardiograms and MRI. Treatment is not warranted in asymptomatic individuals, and restriction of normal physical activities is not needed.
